# Integrated Analysis of Transcriptome and Proteome of the Human Cornea and Aqueous Humor Reveal Novel Biomarkers for Corneal Endothelial Cell Dysfunction

**DOI:** 10.3390/ijms242015354

**Published:** 2023-10-19

**Authors:** Chae-Eun Moon, Chang Hwan Kim, Jae Hun Jung, Young Joo Cho, Kee Yong Choi, Kyusun Han, Kyoung Yul Seo, Hyung Keun Lee, Yong Woo Ji

**Affiliations:** 1 Institute of Vision Research, Department of Ophthalmology, Yonsei University College of Medicine, Seoul 03722, Republic of Korea; cemoon@yuhs.ac (C.-E.M.);; 2 Department of Ophthalmology, Yongin Severance Hospital, Yongin 16995, Republic of Korea; 3 Department of Applied Chemistry, College of Applied Science, Kyung Hee University, Yongin 17104, Republic of Korea; 4 The Yonsei Eye Clinic, Seoul 06289, Republic of Korea; 5 Department of Ophthalmology, HanGil Eye Hospital, Incheon 21388, Republic of Korea; 6 Department of Ophthalmology, Gangnam Severance Hospital, Seoul 06273, Republic of Korea; 7 College of Pharmacy, Yonsei University, Incheon 21983, Republic of Korea

**Keywords:** corneal endothelial cell dysfunction, aqueous humor, biomarker, proteomics, transcriptomics

## Abstract

Earlier studies have reported that elevated protein levels in the aqueous humor (AH) are associated with corneal endothelial cell dysfunction (CECD), but the details of the underlying mechanism as well as specific biomarkers for CECD remain elusive. In the present study, we aimed to identify protein markers in AH directly associated with changes to corneal endothelial cells (CECs), as AH can be easily obtained for analysis. We carried out an in-depth proteomic analysis of patient-derived AH as well as transcriptomic analysis of CECs from the same patients with bullous keratopathy (BK) resulting from CECD. We first determined differentially expressed genes (DEGs) and differentially expressed proteins (DEPs) from CECs and AH in CECD, respectively. By combining transcriptomic and proteomic analyses, 13 shared upregulated markers and 22 shared downregulated markers were observed between DEGs and DEPs. Among these 35 candidates from biomarker profiling, three upregulated markers were finally verified via data-independent acquisition (DIA) proteomic analysis using additional individual AH samples, namely metallopeptidase inhibitor 1 (TIMP1), Fc fragment of IgG binding protein (FCGBP), and angiopoietin-related protein 7 (ANGPTL7). Furthermore, we confirmed these AH biomarkers for CECD using individual immunoassay validation. Conclusively, our findings may provide valuable insights into the disease process and identify biofluid markers for the assessment of CEC function during BK development.

## 1. Introduction

Corneal endothelial cells (CECs) are located on the posterior surface of the cornea and form a single layer of hexagonal cells. This layer is indispensable for corneal clarity due to its critical role in maintaining the appropriate corneal hydration status [1,2,3]. Unlike epithelial cells, CECs are severely limited in their ability to regenerate following damage [4,5]. Thus, damage to CECs resulting in a loss of barrier integrity, solute transporter dysfunction, or a significant decrease in cellular density leads to visual impairment due to a cloudy cornea [6].

CEC dysfunction (CECD) contributes to bullous keratopathy (BK), which is caused by an insufficient number and density of CECs, eventually resulting in vision loss. At present, the treatment for CECD is the transplantation of healthy CECs; however, the supply of donor corneas is globally limited [7,8]. Although it is well known that conditions such as uveitis and diabetes accelerate CEC loss, the detailed underlying mechanism as well as specific biomarkers for CECD remain elusive [9,10,11]. Evaluating the morphological characteristics of the CECs is crucial for diagnosing and monitoring patients with corneal disorders. Currently, the predominant approach to assess the risk of CECD involves meticulous analysis of CECs using specular microscopy and in vivo confocal microscopy [12]. However, the accuracy of these methodologies is not guaranteed, and they come with a high cost. This underscores the urgent need to identify disease-specific markers for early detection. Research into easily accessible biofluids, particularly the aqueous humor (AH), could offer significant insights [13].

**Figure 1:**
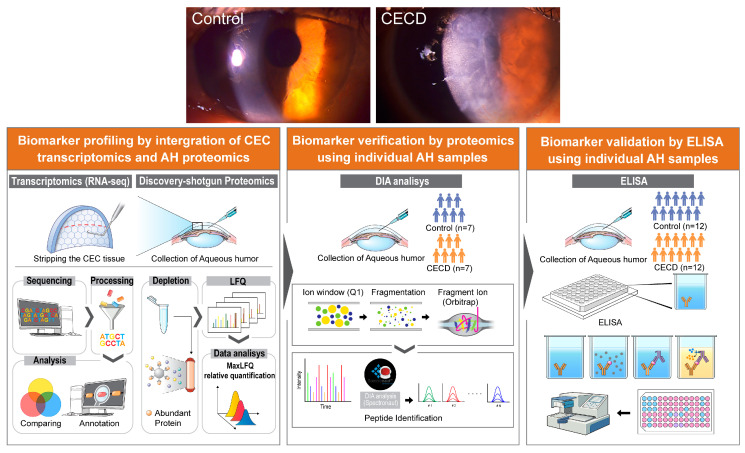
Schematic representation of experimental design for profiling, verification, and validation of the aqueous humor biomarkers for corneal endothelial cell dysfunction. Corneal endothelial cell (CEC) layers were obtained for transcriptome analysis from patients with corneal endothelial cell dysfunction (CECD) after surgical stripping during Descemet membrane endothelial keratoplasty (n = 5). Normal CECs were obtained from donor corneas (n = 5). For label-free quantitative discovery shotgun proteomics, aqueous humor (AH) samples were simultaneously obtained from patients with CECD as well as from patients with cataracts as controls (n = 5/group). After biomarker profiling via integration of CEC transcriptomics and AH proteomics, verification was performed via data-independent acquisition (DIA) proteomic analysis using additional individual AH samples (n = 7/group). Finally, biomarker candidates were validated via enzyme-linked immunosorbent assay (ELISA) using additional individual AH samples (n = 12/group).

AH is a pure intraocular biofluid which remains in direct contact with the inside of the eye. Accordingly, it has a unique advantage over plasma and other fluids in its ability to reflect biochemical alterations of the eye including the posterior cornea [9,14,15]. In particular, changes in the protein content of AH can reflect the condition of CECs, as the area where CECs come into contact with AH within the eyeball accounts for more than 60% of total AH contact area [16]. Furthermore, CECs can secrete their metabolic products directly into the AH [14,16,17]. As AH also mediates immune responses and helps modulate ocular cell proliferation, differentiation, and wound healing, it may contribute to the regulation of CEC properties [18,19,20,21]. Nevertheless, most of the studies that have conducted proteomic analysis of AH have focused on glaucoma or retinal disorders [17,22,23,24,25,26]. Recently, a number of studies have reported that immune response in AH can increase oxidative stress and cause senescence of CECs, leading to CECD or graft failure [27,28,29]. In the present study, we aimed to investigate protein markers in AH directly associated with the changes to CECs, as AH can be easily obtained for analysis. We carried out an in-depth proteomic profiling of patient-derived AH, as well as RNA-sequencing of CECs from the same patients with CECD. Furthermore, we confirmed AH biomarkers for CECD using individual data-independent acquisition (DIA) verification and immunoassay validation (Figure 1). Our findings may provide valuable insights into the disease process as well as diagnostic targets for CECD.

## 2. Results

### 2.1. Transcriptomic Analysis of Dysfunctional CECs

**Figure 2:**
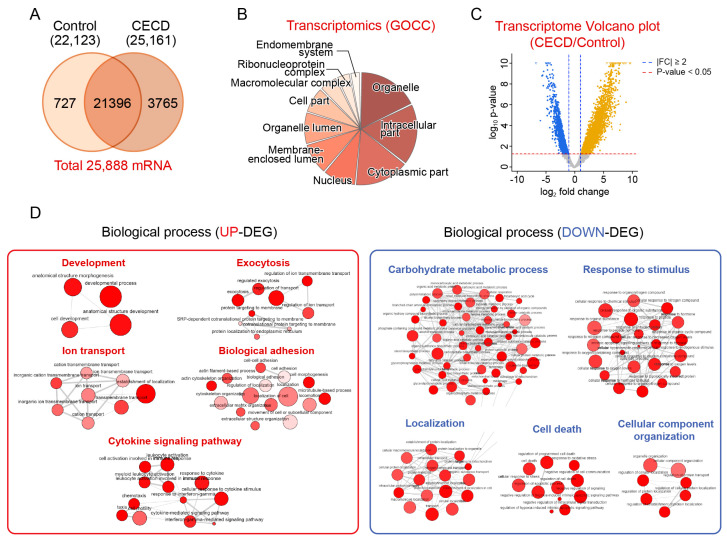
Transcriptomic analysis of dysfunctional human corneal endothelial cells. (**A**) Venn diagram showing total mRNAs from corneal endothelial cells (CECs) in controls and patients with corneal endothelial cell dysfunction (CECD) (n = 5/group). (**B**) Pie chart showing the top 10 significantly enriched terms for gene ontology cellular components (GOCC). (**C**) Volcano plot displaying the difference in gene expression of CECs between two groups. (**D**) Representative gene ontology biological process (GOBP) network and reactome pathway analysis using upregulated (UP-) and downregulated (DN-) differentially expressed genes (DEGs).

A total of 22,123 and 25,161 mRNAs were identified in CECs from the CECD and control groups, respectively. Gene ontology cellular component (GOCC) analysis revealed that the mRNAs identified in the CECs were particularly involved in the intracellular region, organelles, and cytoplasmic parts (Figure 2B). To identify specific changes to gene expression during CECD, we compared gene expression between CECD samples and controls. A total of 2685 upregulated differentially expressed genes (UP-DEGs) and 1832 downregulated differentially expressed genes (DN-DEG) were identified in CECD compared to controls (Figure 2C and Table S1). Multidimensional scaling analysis revealed a clear spatial distance between the two groups with 55% for component 1 (Figure S1).

To gain insight into the functional characteristics of DEGs in CECs, we analyzed the gene ontology biological process (GOBP) network and reactome pathway. UP-DEGs were mainly involved in five categories, namely, the cytokine signaling pathway, biological adhesion, ion transport, development, and exocytosis (Figure 2D and Table S2A). DN-DEGs were also mainly enriched in five categories, namely, carbohydrate metabolic process, localization, cellular component organization, cell death, and response to stimulus (Figure 2D and Table S2B).

### 2.2. Global Proteome Profiling of AH from Patients with CECD

**Figure 3:**
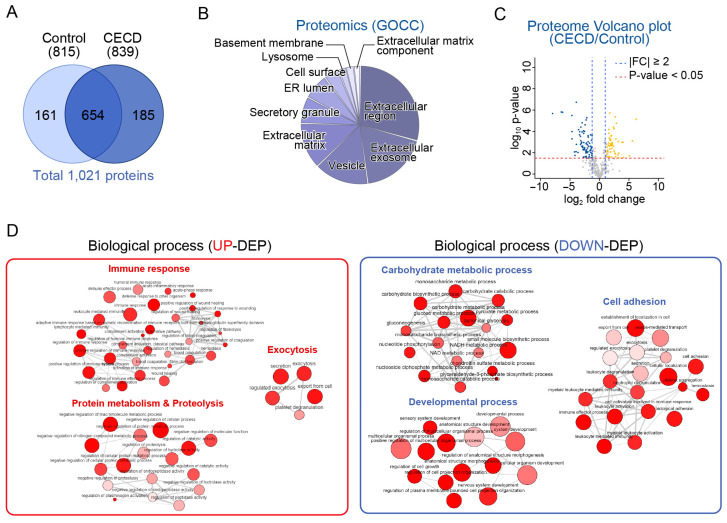
Global proteome profiling analysis of aqueous humor in corneal endothelial cell dysfunction. (**A**) Venn diagram showing total proteins of aqueous humor (AH) from the control and CECD group (n = 5/group). (**B**) Pie chart shows the top 10 significantly enriched terms for gene ontology cellular components (GOCC). (**C**) Volcano plot displaying the difference in protein expression in AH between two groups. (**D**) Representative gene ontology biological process (GOBP) network and reactome pathway analysis using upregulated (UP-) and downregulated (DN-) differentially expressed proteins (DEPs).

To identify proteins altered in pathological AH, we conducted proteomic analysis using patient-derived AH. In total, 839 proteins and 815 proteins were identified in the CECD and control groups, respectively. GOCC functional enrichment analysis revealed that identified AH proteins were mostly involved in the extracellular region, extracellular exosome, membrane-bounded vesicle, extracellular matrix, and secretory granule (Figure 3B). A total of 51 upregulated differentially expressed proteins (UP-DEPs) and 78 downregulated differentially expressed proteins (DN-DEPs) were identified (Figure 3C and Table S3).

GOBP analysis revealed that UP-DEPs were mainly involved in three categories, namely, immune response, proteolysis, and exocytosis (Figure 3D and Table S4A). Immune response was the term most enriched with significantly UP-DEPs, including cystatin S (CST4), lipocalin 1 (LCN1), CD163, TIMP1, ANGPTL7, and FCGBP. DN-DEPs were mainly involved in three categories, namely, carbohydrate metabolic process, developmental process, and cell adhesion (Figure 3D and Table S4B). As compared to changes in mRNA expression in CECs, it was confirmed that immunological responses such as cytokine signaling were significantly upregulated. In contrast, a commonly downregulated pathway at both the mRNA and protein levels was the carbohydrate metabolic process.

### 2.3. AH Proteome Alterations Are Associated with CEC Transcriptome Changes in CECD

**Figure 4:**
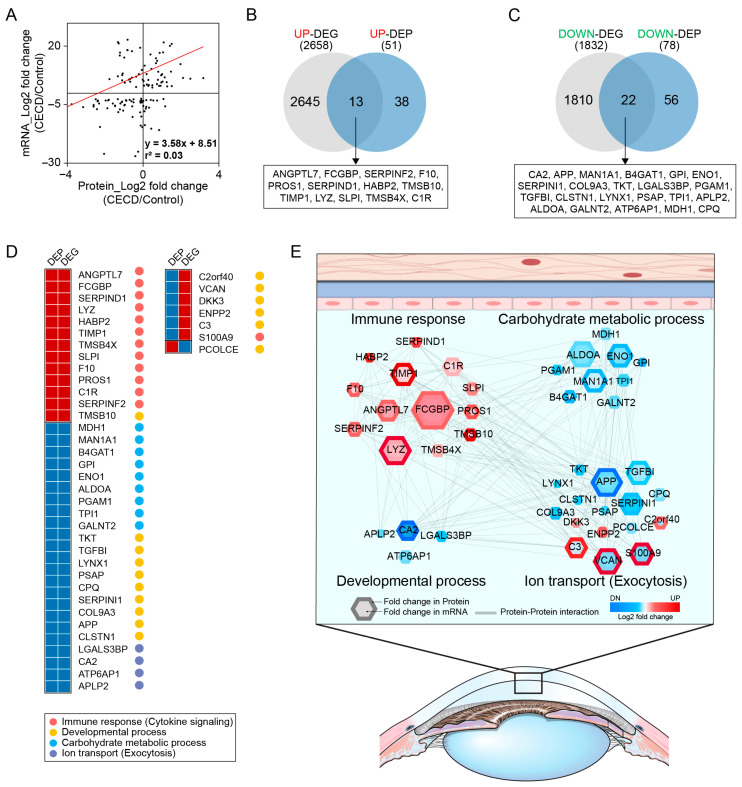
Aqueous humor proteome alterations associated with corneal endothelial transcriptome changes in corneal endothelial cell dysfunction. (**A**) Correlation between the level of expression of aqueous humor (AH) proteins and corneal endothelial cell (CEC) mRNAs in the corneal endothelial cell dysfunction (CECD) group as compared to the control (n = 5/group). (**B**,**C**) The number and list of upregulated and downregulated AH differentially expressed proteins (DEPs), which showed the same pattern of differential expression as observed for their respective transcripts in CECs. (**D**) Gene ontology biological processes of AH DEPs correlated to CEC differentially expressed genes (DEGs). (**E**) Protein–protein interaction network model showing significantly enriched biological processes in AH for CECD. The colors of the nodes represent highly increased (red) or decreased (blue) proteins in CECD. The connection between nodes (grey lines) indicates either a regulatory role or physical interaction between proteins. Large nodes represent a high degree of connectivity with other proteins.

There was a significant correlation between AH protein and CEC mRNA expression under CECD (Figure 4A).

As demonstrated by the Venn diagram in Figure 4B, there were 13 commonly upregulated DEGs and DEPs, namely, ANGPTL7, FCGBP, SERPINF2, F10, PROS1, SERPIND1, HABP2, TMSB10, TIMP1, LYZ, SLPI, TMSB4X, and C1R. Interestingly, all of them were related to the immune response (Figure 4C). In contrast, we found 22 proteins that were downregulated at both the mRNA and protein levels. These are listed in the heat map and include MDH1, MAN1A, B4GAT1, GPI, ENO1, ALDOA, PGAM1, TPI1, GALNT2, TKT, TGFBI, LYNX1, PSAP, CPQ, SERPINI1, COL9A3, APP, CLSTN1, LGALS3BP, CA2, ATP6AP1, and APLP2. Of these, 41% were related to the carbohydrate metabolic process, 41% to the developmental process, and 18% to ion transport (Figure 4C). There were seven proteins exhibiting conflicting trends between their mRNA and protein levels. Among them, six proteins, including C2orf40, ENPP2, C3, VCAN, S100A9, and DKK3, were increased at the mRNA level in CECs yet decreased at the protein level in AH. In contrast, *POLCE* was decreased at the mRNA level, whereas its protein levels in AH were increased (Figure S2A,B).

To provide further insight into the physiological processes underlying CECD, protein–protein interaction network analysis was carried out based on 32 DEPs and DEGs, which are commonly altered (Figure 4D). As shown in Figure 4D, four pathways were significantly enriched, namely, immune response, carbohydrate metabolic process, developmental process, and ion transport. Of note, 13 upregulated proteins, including TIMP1, ANGPTL7, FCGBP, LYZ, and C1R, participate in diverse immune responses, acting as aberrant activators of innate immune system in CECD. In particularly, TIMP1, ANGPTL7, FCGBP, and LYZ were crucial proteins with the highest centrality within the immune response network. Conversely, commonly downregulated transcripts of CECs and their protein products in AH were enriched in carbohydrate metabolic process (e.g., ALDOA, ENO1, MAN1A1), ion transport (e.g., CA2), and developmental process (e.g., APP, SERPINI1, TGFBI). Similarly, seven molecules, which were inversely altered at the mRNA and protein levels, were identified as having pivotal roles in the developmental process.

### 2.4. Individual AH Analysis for Marker Verification via DIA Proteomics in CECD

**Figure 5:**
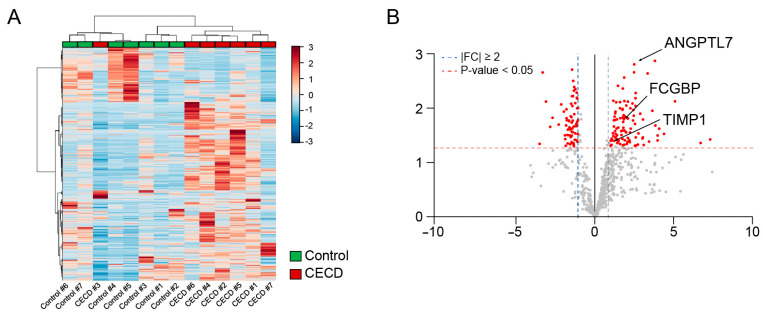

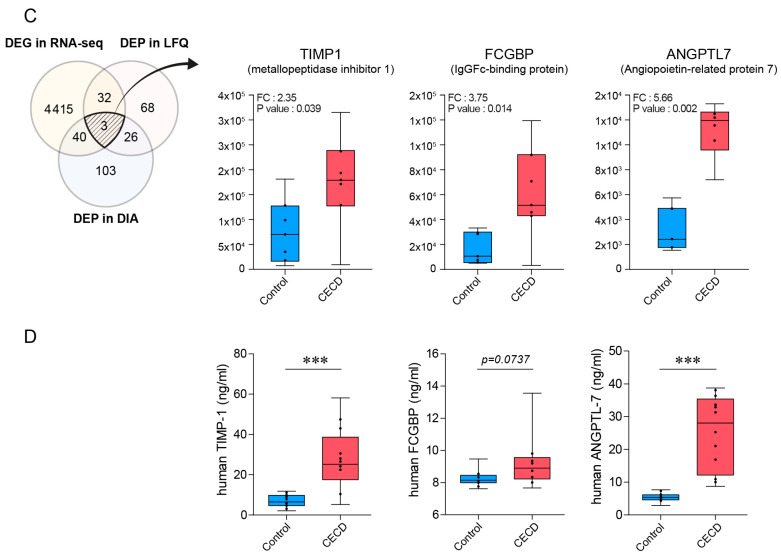
Individual aqueous humor analysis for marker verification via data-independent acquisition proteomics and validation of aqueous humor marker candidates using enzyme-linked immunosorbent assay in corneal endothelial cell dysfunction. (**A**) Hierarchical clustering of differentially expressed proteins (DEPs) in the corneal endothelial cell dysfunction (CECD) group and control group after data-independent acquisition (DIA) proteomic analysis (n = 7/group). (**B**) Volcano plot displaying the difference in protein expression in aqueous humor (AH) between the two groups. (**C**) Three AH proteins were identified as CECD markers based on AH DIA proteomics combined to CEC transcriptomics (RNA-seq) and AH label-free quantitative shotgun proteomics (LFQ) (n = 7/group; two-tailed Student’s *t*-test). (**D**) Validation of candidate marker proteins in the AH of patients with CECD via enzyme-linked immunosorbent assay. Bar graphs represent the mean ± standard error of the mean (n = 12/group; ***, *p* < 0.001 by two-tailed Student’s *t*-test).

To verify altered protein expression, we performed individual DIA analysis of CECD and control groups. A heat map of protein expression profiles indicated that AH samples of the CECD and control groups were mostly separated into two clusters (Figure 5A). As demonstrated by the volcano plot in Figure 5B, there were 106 significantly increased proteins and 66 significantly decreased proteins in CECD patient samples as compared to controls. DEPs obtained via label-free quantitative shotgun proteomics (LFQ) profiling and DIA analysis are presented in the Venn diagram (Figure S3 and Table S5). A total of 22 proteins were found to be increased in both analyses. Further, three targets were increased at both the transcript and protein levels, namely, TIMP1, FCGBP, and ANGPTL7, whereas there were no commonly decreased targets (Figure 5C).

### 2.5. Validation of AH Marker Candidates for CECD

To validate marker candidates identified in the proteomics analyses, we performed enzyme linked immunosorbent assay (ELISA) to quantify AH protein concentrations of the three biomarkers TIMP1, ANGPTL7, and FCGBP. In accordance with proteomics results, ELISA revealed that the concentration of TIMP1 and ANGPTL7 in AH was significantly increased in an independent group of patients with CECD as compared to the control group (Figure 5D). In addition, FCGBP was slightly increased in the CECD group compared to the control group, although these associations were not significant (*p* = 0.0737). These data indicated that TIMP1, ANGPTL7, and FCGBP may serve as ocular biofluid biomarkers, reflecting dysfunction of CECs.

## 3. Discussion

As AH is a good liquid biopsy source for ocular disease, various earlier works have focused on the analysis and identification of specific proteins or metabolites in AH for conditions such as primary open angle glaucoma [23,30], uveal melanoma [22], and pseudo-exfoliation syndrome [26,31]. However, there has been no confirmative evidence for disease-specific biomarkers, which can be used for diagnosis, prognosis, or treatment monitoring. Further, few studies have been performed for the identification of DEPs in CEC disease [27]. Although tools such as specular microscopy, in vivo confocal biomicroscopy, and slit-lamp facilitate the morphological assessment of the cornea, there remains a gap in methodologies for determining the functional status of CECs. Biomarkers are therefore necessary for the evaluation of CEC status and treatment, as well as for the prevention of further CEC damage and progress to end-stage CEC failure.

Herein, via transcriptomic and proteomic analyses, we measured mRNA expression and protein levels in CECs and AH, respectively, comparing these between patients with CECD and controls. The analyses provided the data basis for identifying biomarkers for CEC function. Employing “OMICS” usually results in the identification of hundreds of markers [32,33]. Thus, it is not easy to find and confirm reliable markers via a single OMICS analytical approach. To identify markers more precisely and reliably, we decided to use both transcriptomics and proteomics. Based on data from both analyses, 13 markers were upregulated both at the gene and protein levels, leading us to some interesting findings.

First, endopeptidase inhibitors and protease inhibitors such as ITIH3, SERPINF2, and FeTuB were significantly increased in the AH of patients with CECD. Regulation of proteolysis is vital for maintaining the transparency of ocular tissues, as the aggregation of proteolytic products and accumulation of unprocessed protein, acting as antichaperones, may lead to functional impairment [34]. In agreement with our findings, Kliuchnikova et al. reported that endopeptidase inhibitors and protease inhibitors are significantly enriched in the AH of patients with glaucoma [26].

Second, among the 13 upregulated markers, many were already reported in the AH of patients with glaucoma (e.g., TIMP1, FCGBP). Although the patients included in the present study did not exhibit intraocular pressure (IOP) elevation or glaucoma, it is well known that glaucoma may affect CEC function and vice versa [35,36,37]. To validate OMICS evidence, we performed an ELISA assay for TIMP1, FCGBP, and ANGPTL7 in AH, and we found that all three markers were significantly elevated in samples from patients with CECD (Figure 5).

TIMP1 expression has also been reported in several corneal disease conditions, including BK and keratoconus [38]. Interestingly, TIMP1 was found to be an essential factor for CEC migration and proliferation [39]. Considering the general function of TIMP1, which is the inhibition of matrix proteinase and enhancement of extracellular matrix deposition, TIMP may inhibit normal deposit-clearing activities of CECs, leading to the accumulation of metabolic waste and eventual dampening of normal CEC functions. 

ANGPTL7 was profoundly increased at both the gene and protein levels in CECs and AH from patients with CECD, respectively. Earlier publications reported that ANGPTL7 is closely related with several pathologic conditions, including increased IOP [40,41] and corneal vascularization [42]. Considering the known role of ANGPTL7 in the Wnt/β-catenin signaling pathway and matrix protein assembly, accumulation and disposal of extracellular matrix protein are closely related to BK pathophysiology. 

FCGBP is an immunoglobulin FC-binding protein known to be involved in immunity and inflammation. Sharma et al. also reported that FCGBP plays an important role in inflammatory processes associated with glaucoma [23]. Considering the above-described findings from earlier studies, we identified biomarkers through two OMICS approaches and validated their levels in AH from patients with CECD by ELISA. Thus, the current results strongly supported the specificity of these markers for CECD. 

Apart from the upregulated genes, 22 targets were identified as downregulated at both the transcriptional and protein levels, labeled as DN-DEPs and DN-DEGs, respectively. Most markers downregulated in CECD were associated with metabolic processes. Downregulation of carbohydrate metabolic process proteins such as phosphoglycerate mutase 1 (PGAM1), mannosidase alpha class 1A member 1 (MAN1A1), and beta-1,4-glucuronyltransferase 1 (B4GAT1) may indicate impaired antioxidant defense as well as impaired energy production for Na-K+ ATPase in the AH of patients with CECD. With regard to the metabolic aspect, decreased levels of GPI, ENO1, PGAM1, and ALDOA were indicative of a lower glycolytic capacity in patients with BK as compared to controls. In addition, Wnt pathway antagonists such as PEDF and DKK3 were downregulated in patients with BK, which is indicative of a possible role of Wnt signaling in the pathophysiology of CECD. It is plausible to presume that the iron transport might be reduced in BK. As Na-K+ ATPase enzymes are abundant in CECs, we initially considered the possibility that the non-membrane-bound Na-K+ ATPase in AH could be a CECD marker. However, Na-K+ ATPase was not differentially expressed. Of note, other ion transporters, namely, CA2 and ATP6AP1, were found to be decreased in both AH and CECs of patients with BK. 

There are some limitations of the current study. First, even though we selected cases without other ocular pathologies, the patient group could not be homogeneous. Secondly, our initial global profiling relied on technical replicates combined into pooled samples. This decision stemmed from challenges related to the limited protein content and the inherent scarcity of samples. While this method offered certain advantages, we recognized its potential drawbacks. To address this and ensure a more comprehensive understanding, we subsequently employed biological replicates in our DIA proteomics and ELISA experiments. Third, we chose markers that were differentially expressed at both the transcriptional and protein levels. As proteomics generally yields a smaller number of markers compared to transcriptomics, some novel markers, which might be specific to CECD and were identified exclusively in the transcriptomic analysis, were filtered out. However, we are confident that the more sensitive and reliable proteomic method (i.e., MaxLFQ, DIA) addresses this potential limitation.

In conclusion, using the compiled data from both mRNA sequencing and proteomics, we identified several novel AH markers indicative of CEC dysfunction in patients with BK using the compiled data from both mRNA sequencing and proteomics. We believe that our protein markers will provide a basis for an improved understanding of CECD in BK development. As there are no functional assays for CECs in the clinic, our AH biomarkers may be useful as liquid biopsy targets for the assessment of CEC function in patients with BK.

## 4. Materials and Methods

### 4.1. Participants and Sample Collection

This study was conducted in accordance with the ethical principles specified in the Declaration of Helsinki and Good Clinical Practice Guidelines. It was approved by the Institutional Review Board of Yonsei University College of Medicine (Seoul, Republic of Korea; IRB No. 3-2017-0361) before study initiation, and written informed consent was obtained from each patient prior to participation in the study. Participants had no other corneal disease except BK from CECD. We excluded patients younger than 20 years; those with any ocular history even in the other eye, such as ocular surgery, ocular trauma, ocular infection, allergy, ocular inflammation (i.e., uveitis), glaucoma, or retinal diseases including macular edema; patients using topical eye drops other than artificial tears; contact lens wearers; and those with any systemic disease including autoimmune disease, diabetes, and cerebrovascular disease. Detailed demographic data are summarized in Table 1. We simultaneously collected CEC layers and AH biofluid from five patients with CECD during Descemet’s membrane endothelial keratoplasty (DMEK) (Figure 1). Normal CECs were obtained from sex-matched domestic donor corneas with postmortem time less than 24 h. The procedure was also approved by the IRB (No. 3-2017-0361) and written informed consent was obtained from the donor’s family. And normal AH was obtained from age/sex-matched patients with cataract during surgery. Approximately 150 μL of AH was sampled from the anterior chamber during surgery. All samples were stored at −80 °C until measurement.

### 4.2. RNA Preparation and Sequencing of CECs

From the remnant donor tissue after corneal transplantation, human CEC layers for the control group were harvested according to the well-known procedure used for preparation of the donor tissue for DMEK [43,44,45]. Briefly, the CEC monolayer, along with its Descemet’s membrane (DM), was carefully peeled away from the posterior stroma of the cornea with gentle manual dissection after mounting the donor corneoscleral rim on a fixation device with the endothelial side up. For CECs of the CECD group, the diseased recipient CEC monolayer with DM was stripped with a descemetorhexis during the DMEK operation as described previously [46]. The CEC layers were then lysed in 700 μL of QIAzol lysis reagent (Qiagen, Valencia, CA, USA), and stored at −80 °C until used for experiments. Preparation of the libraries and RNA sequencing analysis was conducted by Macrogen, Inc. (Seoul, Republic of Korea). cDNA was prepared using SMARTer procedure with the SMART-Seq v4 Ultra Low Input RNA Kit (Takara Bio USA, San Jose, CA, USA) for Illumina Sequencing. Human corneal endothelium mRNA profiles of normal and CECD were generated via deep sequencing in triplicate, using NovaSeq 6000 (Illumina, San Diego, CA, USA). We preprocessed the raw reads obtained from the sequencer to remove low-quality reads and adapter sequences before analysis. High-quality reads were then aligned to the Homo sapiens (GRCh38) reference genome using HISAT2 v2.1.0. The reference genome sequence of Homo sapiens (GRCh38) and annotation data were downloaded from National Center for Biotechnology Information. Transcript assembly of known transcripts was processed by StringTie v1.3.4d. Based on the results, expression abundance of transcripts was calculated as read count or fragments per kilobase of exon per million fragments mapped (FPKM) value per sample. The relative abundance of transcripts was measured in read count using StringTie. For the DEG set, hierarchical clustering analysis was performed using complete linkage and Euclidean distance as measures of similarity.

### 4.3. Sample Preparation for Proteomic Analysis

Five individual AH samples were combined in equal volumes to create a pooled sample for the initial global profiling using LFQ. To ensure the accuracy and reproducibility of the experimental results, each pooled sample was analyzed in triplicate. An additional seven AH samples from each group were subjected to DIA verification. For each AH protein sample, we eliminated highly abundant proteins, which tend to obscure the detection of potential marker proteins presented at low abundance in AH, using Seppro IgY spin columns (Sigma Aldrich, St. Louis, MO, USA). The protein concentrations were measured in duplicate using a bicinchoninic acid (BCA) protein assay, following the manufacturer’s protocol (Thermo Scientific Pierce, Rockford, IL, USA). Subsequently, 100 μg of total protein samples were digested into peptides using an in-solution digestion method as previously described. Briefly, 10 M urea in 100 mM ammonium bicarbonate was mixed with each sample (*v*/*v*, 1:1), resulting in a final concentration of at least 5 M, and the mixture was incubated for 30 min at room temperature for denaturation. Approximately 10 mM dithiothreitol for reduction and 30 mM iodoacetamide for alkylation were used to denature proteins. Trypsin was added at a 50:1 (*w*/*w*) protein-to-protease ratio and incubated at 37 °C overnight. The activated trypsin reaction was quenched with 0.4% trifluoroacetic acid, and peptides were desalted with a C18 Harvard macro spin column. The resultant peptides were dried and stored at −80 °C.

### 4.4. Quantitative Global Profiling

For LFQ analysis, peptides were re-suspended in 0.1% formic acid in water and analyzed using the Q Exactive orbitrap hybrid mass spectrometer (Thermo Fisher Scientific, San Jose, CA, USA) coupled with the EASY-nLC 1000 system (Thermo Fisher Scientific, Bremen, Germany). Solvents A and B were 0.1% formic acid in water and 0.1% formic acid in acetonitrile, respectively. A 200-min gradient (from 5% to 20% solvent B over 150 min, from 20% to 35% solvent B over 30 min, 80% solvent B for 10 min, and 5% solvent B for 10 min) was used. The peptides were loaded onto a trap column (75 μm × 2 cm, 3 μm, C18, 100 Å) and ionized via an EASY-spray column (50 cm × 75 μm ID) packed with 2-μm C18 particles at an electric potential of 2.0 kV. Full MS scans were acquired in a scan range of 350–2000 Th at a resolution of 70,000 at *m*/*z* 200. The ten most abundant ions were fragmented via data-dependent MS/MS experiments with an isolation window of 2.0 Th and exclusion duration of 30 s and at a normalized collision energy of 27 for higher energy collisional dissociation (HCD). The charge state of 1 was discarded. Maximum ion injection times were 100 ms and 50 ms for full MS and MS/MS scans, respectively. The automated gain control (AGC) target value was set to 1.0 × 10^6^ for both MS and MS/MS scans.

### 4.5. Individual Aqueous Humor Analysis and Data Processing

For DIA analysis, a retention time kit (iRT kit, Biognosys, Schlieren, Switzerland) was used to spike samples at a concentration of 1:20 *v*/*v* and 2 μg of each peptide sample was analyzed us-ing Q-Exactive plus (Thermo Fisher Scientific) equipped with an EASY-nLC 1000 UHPLC System (Thermo Fisher Scientific) using a range of 500–900 *m*/*z* with a resolution of 170,000 at 200 *m*/*z*. The AGC target was set to 1e6 with a 60-ms maximum injection time. Twenty optimal acquisition windows covered a mass range from 500 to 900 *m*/*z*. The normalized collision energy for HCD-MS2 experiments was set to 30%, the AGC target was set at 2 × 10^5^, and the maximum injection time was set to 60 ms. The mass spectrometry proteomics data were deposited to the ProteomeXchange Consortium via the PRIDE [47] partner repository with the dataset identifier PXD023772. The DIA data were analyzed with Spectronaut Pulsar (version 11.0.15038.4.29119, Biognosys) using a search archive spectral library, and the default settings were used for targeted analysis. In brief, a dynamic window for the XIC extraction window and a non-linear iRT calibration strategy were used. Mass calibration was set to local mass calibration. Interference correction on the MS1 and MS2 levels was enabled, removing fragments/isotopes from quantification based on the presence of interfering signals but keeping at least three for quantification. The FDR was set to 1% at the peptide precursor level and 1% at the protein level.

### 4.6. Database Search and Quantitative Analysis

For data-dependent analysis profiling, the MS2 spectra were searched with MaxQuant (v. 1.5.7.4) against the Uniprot human database (released in 2017_06, 20,205). Carbamidomethylation of cysteine as a fixed modification and N-acetylation and oxidation of methionine as variable modifications were used for each search. An FDR cutoff of 1% was applied at the peptide spectrum match (PSM) and protein levels. An initial precursor mass deviation of up to 4.5 ppm and a fragment mass deviation of up to 20 ppm were allowed. Protein identification required at least one peptide using the “razor plus unique peptides” setting in MaxQuant. Proteins were quantified using the XIC-based LFQ algorithm in MaxQuant. The “match between runs” option was used for nonlinear retention time alignment. The match time window was 0.7 min, and the alignment time window was 20 min. Before loading LFQ intensity data, hits to the reverse database, contaminants, and proteins identified only by site were eliminated. After loading the data, all triplicate data were grouped separately. All LFQ intensities were transformed to log2 values. Proteins that did not display all values in at least one group were filtered out. Additionally, in cases with a missing value, missing values were replaced via imputation based on the normal distribution (using a width of 0.3 and a downshift of 1.8). To account for nonbiological variability of MS data resulting from many factors, including sample preparation and instrumental biases, intensities were normalized using MaxQuant program.

### 4.7. Enrichment Analysis Using Gene Ontology

A GO search was performed using g-Profiler to explore GOBP and GOCC in CECs and AH associated with CECD. GOBPs with a *p*-value < 0.05 were identified as enriched by DEGs and DEPs. To construct a network depicting enriched processes, GO enrichment analysis results were visualized and interpreted in Cytoscape using its EnrichmentMap tool. To construct the network model for genes commonly altered in DEGs and DEPs, we collected protein-protein interactome information from the STRING version 11 public database. The network model was built using Cytoscape.

### 4.8. Enzyme-Linked Immunosorbent Assay (ELISA)

ELISA was conducted to verify the biomarker candidate proteins identified in transcriptomic and proteomic analyses. An additional 12 AH samples from each group were subjected to ELISA. AH proteins were measured using the human metallopeptidase inhibitor 1 (TIMP1) (DTM100; R&D Systems), angiopoietin-related protein 7 (ANGPTL7), and Fc fragment of IgG binding protein (FCGBP) ELISA Kit (CSB-EL001715HU, CSB-EL008536HU; Cusabio Technology) according to the manufacturer’s instructions [48,49]. Briefly, all samples were brought to room temperature before use and were assayed in duplicate. 50 μL of samples or standard was added into the wells pre-coated with an antibody specific to the antigen (human TIMP-1, human ANGPTL7, or human FCGBP) and incubated for 2 h at room temperature. This was followed by incubation for 2 h with the target antibody conjugates. Substrate solution was added to the samples and was incubated for 30 min. Stop solution was added, and the absorbance of color at 450 nm was measured using a microplate reader (Bio-Rad^®^ Microplate Absorbance Reader, Bio-Rad Laboratories Inc., Hercules, CA, USA). The intra-assay and inter-assay coefficients of variation within and between ELISA tests were <8%. All absorbance results are expressed as nanogram per milliliter.

### 4.9. Statistics

This study was conducted in accordance with the ethical principles specified in the Declaration of Helsinki and Good Clinical Practice Guidelines. It was approved by the Institutional Review Board of Yonsei University College of Medicine (Seoul, Republic of Korea; IRB No. 3-2017-0361) before study initiation, and written informed consent was obtained from each patient prior to participation in the study. Statistical analyses on the clinical data were performed using SPSS version 21.0 (IBM Corp., Armonk, NY, USA). The Kolmogorov–Smirnov test was used to confirm normality of the data. To statistically compare data between groups, we used the Mann–Whitney U test or Wilcoxon signed rank test for non-normally distributed data. In all statistical tests, a *p*-value less than 0.05 was considered statistically significant. Statistical significance of DEGs was determined using the edgeR exact Test and FC, in which the null hypothesis was that no difference exists between groups. DEGs were determined via a threshold *p*-value < 0.05 and values of FC > 2 from Student’s *t*-test. Perseus software (v.1.6.1.1) was used for the statistical and bioinformatics analyses of proteomics data [50]. The selection criteria of DEPs were fold change (FC) > 2 and *p*-value < 0.05 from Student’s *t*-test. Comparisons of mean protein levels as detected by ELISA were performed by using Student’s *t*-test, with *p*-value < 0.05 being significant.

## Figures and Tables

**Table 1:** Demographic characteristics and clinical parameters of patients enrolled in the present study. (**A**). Transcriptome analysis of CECs using RNA-sequencing. (**B**). Global proteome profiling of AH using LFQ analysis. (**C**). Individual analysis of AH using DIA proteomics. (**D**). Validation of AH markers for CECD using ELISA.

(**A**)		
	**Control (Donor) (n = 5)**	**CECD (n = 5)**
Age, y	43.75 ± 6.55	69.25 ± 3.86 *
Sex, n (Female:Male)	2:3	2:3
CEC density, /mm^2^	2998.25 ± 112.87	Not available
Corneal thickness, μm	555.25 ± 5.44	Bullous
(**B**)		
	**Control (n = 5)**	**CECD (n = 5)**
Age, y	61.54 ± 9.22	69.25 ± 3.86
Sex, n (Female:Male)	2:3	2:3
CEC density, /mm^2^	2523.32 ± 114.42	Not available
Corneal thickness, μm	546.50 ± 6.32	Bullous
(**C**)		
	**Control (n = 7)**	**CECD (n = 7)**
Age, y	68.14 ± 6.36	69.42 ± 7.04
Sex, n (Female:Male)	3:4	3:4
CEC density, /mm^2^	2611.32 ± 122.32	Not available
Corneal thickness, μm	548.25 ± 5.23	Bullous
(**D**)		
	**Control (Donor) (n = 12)**	**CECD (n = 12)**
Age, y	60.5 ± 4.19	55.17 ± 12.91
Sex, n (Female:Male)	7:5	7:5
CEC density, /mm^2^	2549.43 ± 103.67	Not available
Corneal thickness, μm	551.14 ± 4.27	Bullous

CEC layers and AH biofluids were simultaneously collected from five patients with CECD. For marker validation, DIA and ELISA experiments were subsequently conducted using different patient sets. Data are presented as mean ± standard deviation. *p*-values for comparisons of clinical values between two groups were determined using Student’s *t* test. *, *p* < 0.05; CECD, corneal endothelial cell dysfunction; CECs, corneal endothelial cells; AH, aqueous humor; LFQ, label-free quantitative shotgun proteomics; RNA-seq, RNA sequencing; DIA, data independent acquisition; ELISA, enzyme-linked immunosorbent assay.

## Data Availability

Data are available via ProteomeXchange with the identifier PXD023772 and NCBI GEO with the dataset identifier GSE176481.

## Supplementary Materials

The following supporting information can be downloaded at: https://www.mdpi.com/article/10.3390/ijms242015354/s1.
