# Supplementary material for: Integrated Analysis of Transcriptome and Proteome of the Human Cornea and Aqueous Humor Reveal Novel Biomarkers for Corneal Endothelial Cell Dysfunction
Source: Int J Mol Sci. 2023 Oct 19;24(20):15354. doi: 10.3390/ijms242015354 (PMC10607268; doi:10.3390/ijms242015354)
Supplement: Supplementary file 1 [file ijms-24-15354-s001.zip › Fig S1.pdf]

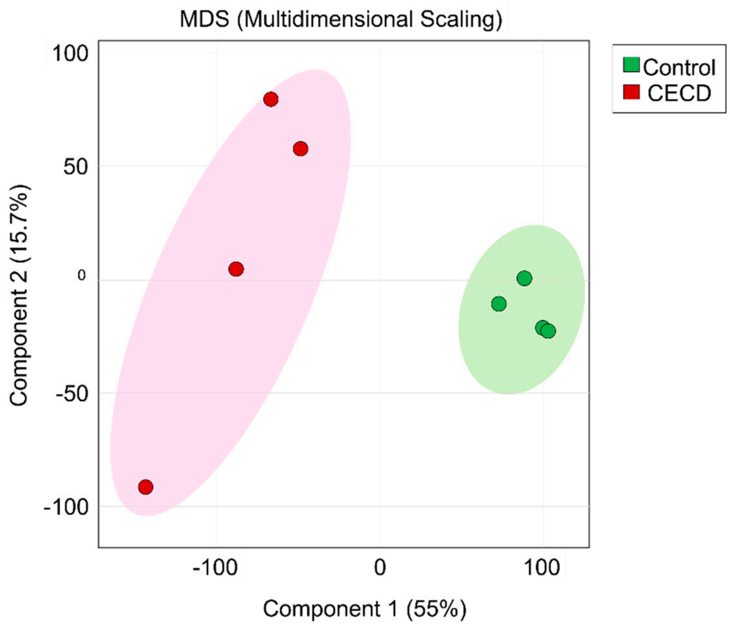

Figure. S1 Multidimensional scaling analysis of differentially expressed genes in dysfunctional corneal endothelial cells. CECD, corneal endothelial cell dysfunction.
