# Supplementary material for: Integrated Analysis of Transcriptome and Proteome of the Human Cornea and Aqueous Humor Reveal Novel Biomarkers for Corneal Endothelial Cell Dysfunction
Source: Int J Mol Sci. 2023 Oct 19;24(20):15354. doi: 10.3390/ijms242015354 (PMC10607268; doi:10.3390/ijms242015354)
Supplement: Supplementary file 1 [file ijms-24-15354-s001.zip › Fig S2.pdf]

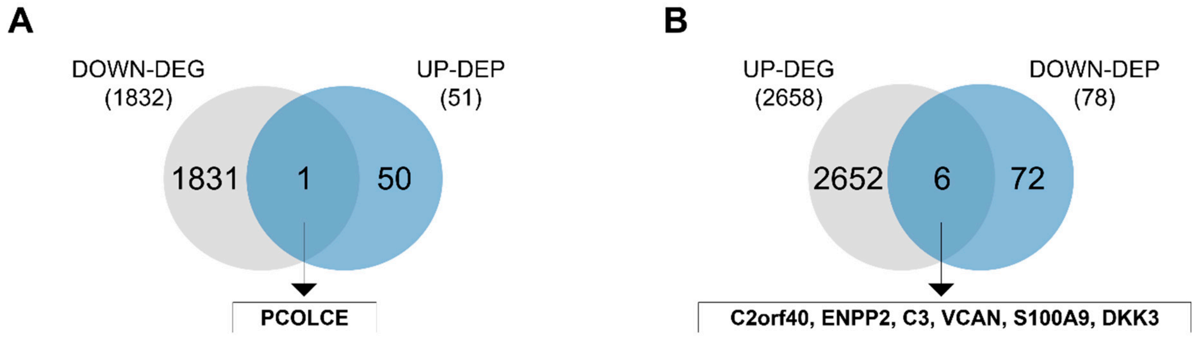

Figure. S2 Aqueous humor proteome alterations inversely associated with corneal endothelial transcriptome changes in corneal endothelial cell dysfunction. (A, B) The number and list of upregulated and downregulated differentially expressed proteins (DEPs) in aqueous humor, which showed conflicting trends of differential expression as observed for their respective genes (DEGs) in corneal endothelial cells.
