# Supplementary material for: Integrated Analysis of Transcriptome and Proteome of the Human Cornea and Aqueous Humor Reveal Novel Biomarkers for Corneal Endothelial Cell Dysfunction
Source: Int J Mol Sci. 2023 Oct 19;24(20):15354. doi: 10.3390/ijms242015354 (PMC10607268; doi:10.3390/ijms242015354)
Supplement: Supplementary file 1 [file ijms-24-15354-s001.zip › Fig S3.pdf]

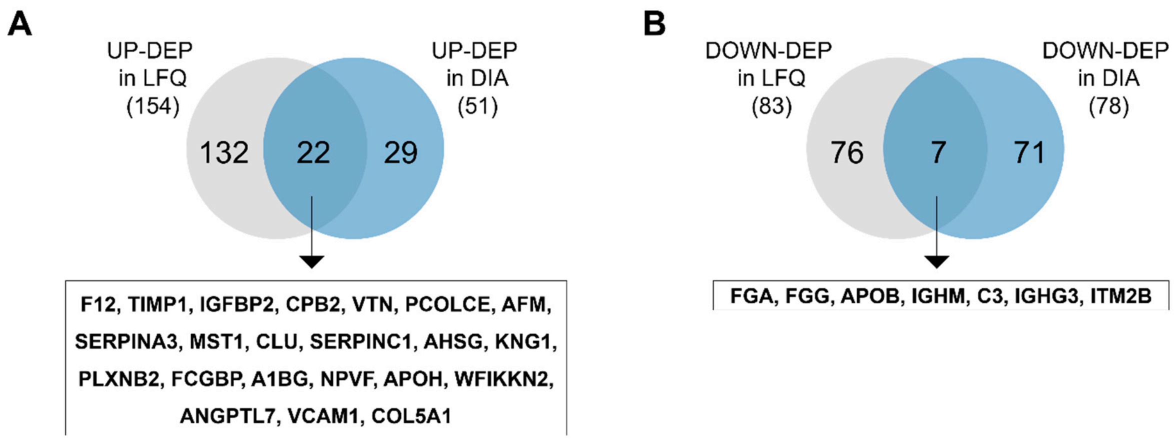

Figure. S3 Aqueous humor proteome obtained by label-free quantification profiling and data-independent acquisition individual analysis in corneal endothelial cell dysfunction. (A, B) The number and list of upregulated and downregulated differentially expressed proteins (DEPs) in aqueous humor with corneal endothelial cell dysfunction, which showed same pattern of differential expression in both label-free quantification (LFQ) profiling and data-independent acquisition (DIA) individual analyses.
